# Brain responses to anticipating and receiving beer: Comparing light, at‐risk, and dependent alcohol users

**DOI:** 10.1111/adb.12766

**Published:** 2019-05-07

**Authors:** Martine M. Groefsema, Rutger C.M.E. Engels, Valerie Voon, Arnt F.A. Schellekens, Maartje Luijten, Guillaume Sescousse

**Affiliations:** ^1^ Executive Board Radboud University, Behavioural Science Institute Nijmegen The Netherlands; ^2^ Executive Board Erasmus University Rotterdam The Netherlands; ^3^ Cambridge University, Behavioural and Clinical Neuroscience Institute Cambridge United Kingdom; ^4^ Radboud University, RadboudUMC Nijmegen The Netherlands; ^5^ Radboud University, Donders Institute for Brain, Cognition and Behaviour Nijmegen The Netherlands; ^6^ Centre de Recherche en Neurosciences de Lyon, INSERM U1028, CNRS UMR5292, PSYR2 Team Lyon France; ^7^ CH Le Vinatier, Service Universitaire d'Addictologie Bron France

**Keywords:** alcohol, brain, reward

## Abstract

Impaired brain processing of alcohol‐related rewards has been suggested to play a central role in alcohol use disorder. Yet, evidence remains inconsistent and mainly originates from studies in which participants passively observe alcohol cues or taste alcohol. Here, we designed a protocol in which beer consumption was predicted by incentive cues and contingent on instrumental action closer to real life situations. We predicted that anticipating and receiving beer (compared with water) would elicit activity in the brain reward network and that this activity would correlate with drinking level across participants.

The sample consisted of 150 beer‐drinking males, aged 18 to 25 years. Three groups were defined based on alcohol use disorders identification test (AUDIT) scores: light drinkers (n = 39), at‐risk drinkers (n = 64), and dependent drinkers (n = 47). fMRI measures were obtained while participants engaged in the beer incentive delay task involving beer‐ and water‐predicting cues followed by real sips of beer or water.

During anticipation, outcome notification and delivery of beer compared with water, higher activity was found in a reward‐related brain network including the dorsal medial prefrontal cortex, orbitofrontal cortex, and amygdala. Yet, no activity was observed in the striatum, and no differences were found between the groups.

Our results reveal that anticipating, obtaining, and tasting beer activates parts of the brain reward network, but that these brain responses do not differentiate between different drinking levels.

## Graphical abstract

In this study, we designed a protocol in which beer consumption was predicted by incentive cues and contingent on instrumental action, closer to real life situations. During anticipation, outcome notification and delivery of beer compared with water, higher activity was found in a reward‐related brain network including the dorsal medial prefrontal cortex, orbitofrontal cortex, and amygdala. Yet no differences were found between light, at risk, or dependent drinkers, suggesting that these brain responses do not differentiate between different drinking levels.

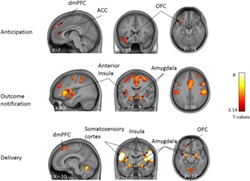

## 1 INTRODUCTION

Excessive alcohol use has been associated with risky behavior and increased mortality,^1^ resulting in a high economic and disease burden worldwide.^2^ Improving prevention and treatment of alcohol use disorders (AUD) requires a better understanding of the underlying mechanisms. At the brain level, disrupted reward processing has been considered as one of the key mechanisms contributing to AUD and more generally to addictive behaviours.^3^, ^4^, ^5^, ^6^, ^7^ Most studies that have probed the reactivity of the brain reward network in AUD have used monetary rewards, showing altered response patterns in the striatum and medial prefrontal cortex (mPFC) (although the direction of these effects remains inconsistent, see Galandra et al and Huys et al^5^, ^8^ for recent reviews). In contrast, fewer studies have investigated the processing of alcohol‐related rewards in AUD. This is important in the light of recent literature arguing that the brain reward network responds differently to addiction versus nonaddiction‐related rewards.^9^, ^10^

Previous studies investigating alcohol‐related reward processing have mostly focused on visual alcohol cue reactivity. Cues that have been repeatedly paired with alcohol use elicit wanting responses resulting from the activation of the brain reward network, including the ventral striatum (VS), amygdala, mPFC, and orbitofrontal cortex (OFC).^7^, ^11^, ^12^ However, as revealed by a recent meta‐analysis, reward‐related brain responses to alcohol cues do not seem to distinguish nondependent from dependent alcohol users,^11^ challenging the importance of the wanting component in explaining AUD. Yet, the studies included in this meta‐analysis have a few limitations. First, most of them have examined brain responses to alcohol pictures that are not followed by actual alcohol consumption and thus presumably do not carry the same incentive value as in real life. In order to investigate whether conditioned alcohol cues elicit exaggerated brain responses in AUD, it is important to ensure that these cues are predictive of real alcohol consumption. Second, it is noteworthy that many cue reactivity studies in dependent alcohol users did not include a control group or had relatively low sample sizes (see Table 1 in Schacht et al^11^), thereby limiting the ability to detect group differences.

**Table 1:** Sample characteristics

	Light Drinkers (n = 39)	At‐risk Drinkers (n = 64)	Dependent Drinkers (n = 47)	Statistics
Age (years)^a^	22.19 ± 1.77	22.10 ± 1.92	22.62 ± 1.73	*F*_(2,147)_ = 1.337, *P =* .266
Education level^b^	70% high, 20% middle, 10% low	76% high, 22% middle, 2% low	79% high, 21% middle, 0% low	*X* ^2^_(4)_ = 7.762, *P* = .101
AUDIT^c^	5.51 ± 1.46	10.95 ± 1.77	21.04 ± 3.44	*F*_(2,147)_ = 488.591, *P* < .001
Weekly drinking^d^	5.88 ± 4.40	13.60 ± 5.11	34.35 ± 9.81	*F*_(2,147)_ = 210.622, *P* < .001
Smoking currently^e^	7.7%	14.1%	27.7%	*X* ^2^_(1)_ = 6.767, *P* = .034

*Note*. Mean ± SD.

Abbreviation: AUDIT, alcohol use disorders identification test.

a Age range 18 to 26 years at first test session.

b Charaterized as low, middle, or high according to the Dutch education system.

c Alcohol use disorder identification test,^13^ range 1 to 29.

d Number of alcoholic drinks based on a 7‐day timeline follow‐back,^14^ range 0 to 71.

e One‐item yes/no question.

More recently, a few studies have examined brain responses to tasting alcohol, investigating the hedonic or “liking” properties of alcohol.^15^, ^16^, ^17^, ^18^, ^19^, ^20^ These studies have revealed that compared with soft drinks, alcohol elicits activity in the reward‐related brain network, in particular in the VS and mPFC among heavy drinking (young) adults (but see Cservenka et al^21^). Importantly, moderate to strong correlations were found between activation in these regions and the severity of alcohol use problems,^16^, ^17^ suggesting that reward‐related brain responses to the taste of alcohol may represent a neurobiological marker of AUD. Yet, these studies have employed passive tasting paradigms in which alcohol was administered in a fully predictable manner and independently of any instrumental action (but see Oberlin et al^22^). This may provide an incomplete picture of alcohol reward processing in AUD, given that reward‐related brain activity is heavily dependent on both the unpredictability of the reward^23^ and the requirement for instrumental action.^24^, ^25^ This is all the more important as brain activity in response to drugs of abuse is thought to depend on whether these drugs are received passively or following contingent action.^26^

In this study, we aimed to investigate whether problematic alcohol use is associated with abnormal reward‐related brain responses to anticipating, obtaining, and tasting beer, while addressing the limitations of previous studies. To this aim, we used functional Magnetic Resonance Imaging (fMRI) combined with a novel task design inspired by the monetary incentive delay task.^27^ This task that we refer to as the beer incentive delay (BID) task used alcohol instead of monetary rewards. Specifically, participants were exposed to abstract cues predicting the delivery of either beer or water (latter being used as a control condition) and had to react fast enough to a visual target in order to receive the predicted reward in the mouth via a tube. Brain responses reflecting motivation or “wanting” were measured during the period preceding the motor action (cue + delay), while brain responses reflecting pleasure or “liking” were measured at the time of reward outcome notification and reward delivery, separately. Importantly, we recruited a large cohort of 150 participants spanning the whole spectrum from light to dependent drinkers. In order to validate our novel task design, we first tested whether beer anticipation, outcome notification, and delivery would elicit higher brain responses in the reward‐related network compared with water. Then, we hypothesized that these brain responses, in particular in the VS, would differentiate light, at‐risk, and dependent alcohol drinkers.

## 2 MATERIAL AND METHODS

### 2.1 Participants

Participants were recruited via flyers and online advertisements. Potential participants initially completed an online screening to assess their eligibility to participate (see detailed flow‐chart in Figure S1). Inclusion criteria at this stage were (a) age 18 to 25 years, (b) being male, and (c) drinking beer. Exclusion criteria were Magnetic Resonance Imaging (MRI) contraindications and a history of brain injury. Participants were further categorized into three groups—light, at‐risk, and dependent drinkers—based on two self‐report measures collected during the initial online screening, as well as the Diagnostic and Statistical Manual (DSM‐IV) criteria for alcohol dependence assessed later during an onsite clinical interview. The self‐report measures assessed the level of alcohol‐related problems (AUD identification test [AUDIT]^13^ and the heaviness of drinking (number of alcoholic drinks per week). We used the recommended cut‐offs for AUDIT scores,^28^ ie, score less than 8 for light drinkers, score between 8 and 15 for at‐risk drinkers, and score greater than 15 for likely dependent drinkers. To further delineate the likely dependent drinkers from the two other groups, we required them to drink more than 22 alcoholic drinks per week (ie, to show a pattern of heavy and excessive drinking behavior as defined by Ihssen et al and Monshouwer et al^29^, ^30^ while the light and at‐risk drinkers had to consume less than or equal to 22 alcoholic drinks per week. Finally, to confirm the suspicion of alcohol dependence diagnosis in the most severe group, we conducted a later onsite screening for these participants and verified that they met the DSM‐IV criteria for alcohol dependence as assessed with the semi‐structured MINI interview (MINI,^31^ administered by a psychologist in training). All participants participated voluntary, gave written consent, and received a financial compensation of 50 euros (with an additional 10 euros for the MINI interview for the dependent drinkers). The study was approved by the regional ethical committee CMO‐Arnhem‐Nijmegen (# 2014/043).

The initial sample consisted of 166 individuals. Seven individuals were incorrectly included, as they did not meet the combined requirement of drinking less than 22 drinks per week with an AUDIT score between 0 and 15, or drinking more than 22 drinks per week with an AUDIT score greater than 15. The data from these seven participants were discarded before performing any data analysis. Six participants further dropped out during data collection. Finally, the data from one participant were missing because of technical problems with the pumps delivering the drinks, and data from two participants were excluded because of excessive head motion in the scanner (greater than 3 mm). Characteristics of the final sample (n = 150) are presented in Table 1. The light drinkers (n = 39), at‐risk drinkers (n = 64), and dependent drinkers (n = 47) were matched for age and education, and none of them was seeking treatment for their alcohol use. The groups differed in alcohol consumption levels and smoking status.

### 2.2 Procedure

Following the screening, participants completed three data collection sessions about 1 week apart: two behavioural sessions in a bar lab and one fMRI session (data from the bar lab will be reported elsewhere, see Table S1 for a complete overview of the data collected in each session). FMRI data acquisition took place between 4:00 pm and 10:00 pm, coinciding with typical drinking hours. Participants were asked to abstain from drinking alcohol in the 24 hours preceding testing, as verified using a breath analyzer. Participants performed two tasks in the scanner, including the BID task in which participants could earn sips of beer and water (see below). Participants consumed a glass of water before scanning to homogenize the level of thirst across participants. After scanning, a breath analyzer was again used to check whether the blood‐alcohol‐levels (BACs) were below the legal .05 limit before participants were allowed to leave.

### 2.3 BID task

**Figure 1:**
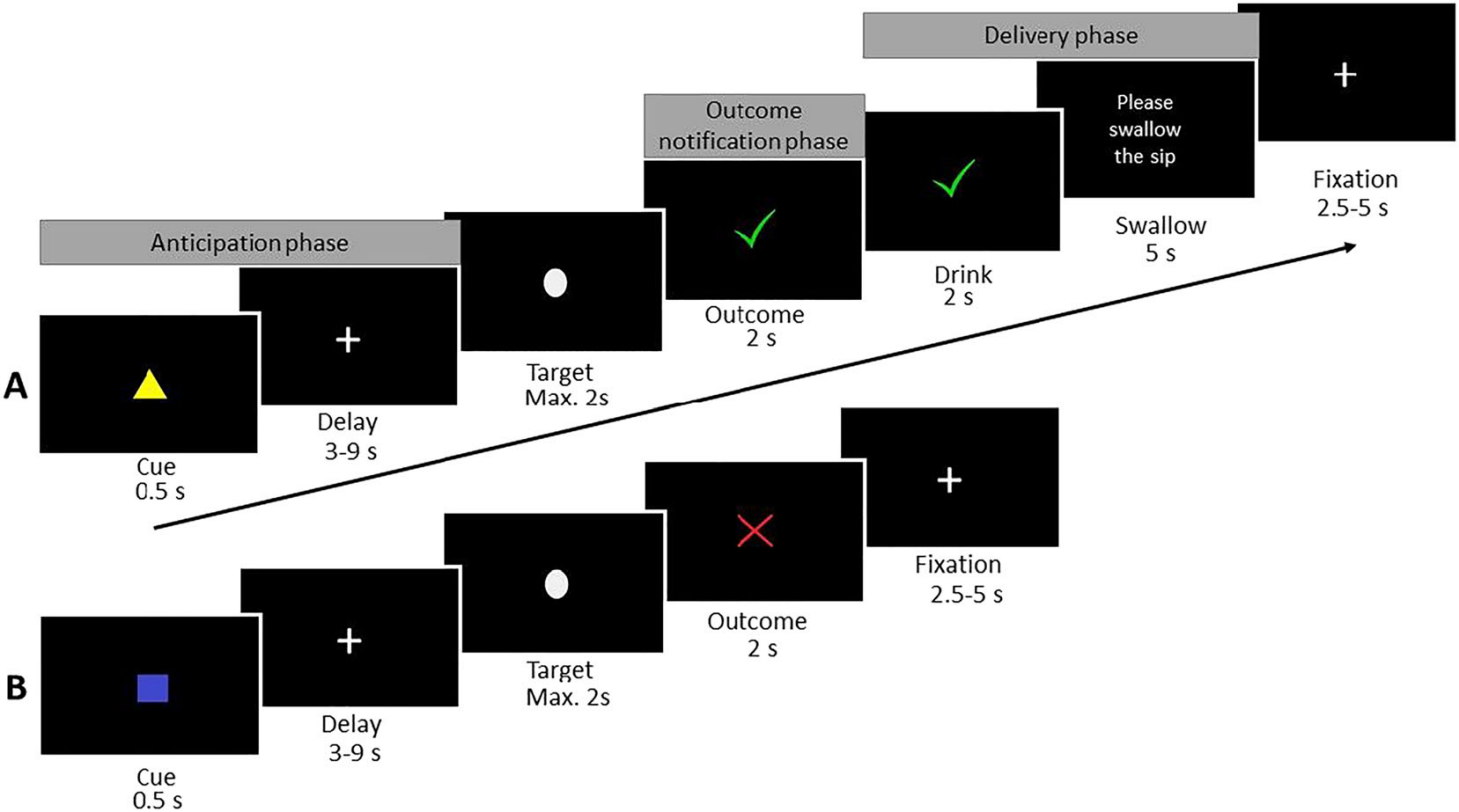
Beer incentive delay task. A, a correct beer trial; B, an incorrect water trial

We used a modified version of the monetary incentive delay task,^27^, ^32^ in which the rewards were 3‐mL sips of either chilled beer or water (Figure 1). The sips were delivered using two StepDos 03RC fluid pumps with tubes that were placed in the participants' mouth. In the anticipation phase, participants first saw a cue informing them about the opportunity to earn beer (yellow triangle) or water (blue square) followed by a variable delay materialized by a fixation cross. Then, a visual target appeared, and participants were instructed to respond to it as fast as possible using a button press, both in the beer and water conditions. If the response was fast enough, positive feedback was provided in the form of a green tick (outcome notification phase), followed by the drink delivery in the mouth, and then swallowing (delivery phase). When the response was too slow, a red cross was presented, ending that trial. Both the beer and water conditions consisted of 30 pseudo‐randomized trials. Ten practice trials preceded the task. Reaction times during the practice trials were used to tailor task difficulty to each individual (by adjusting the time limit for responding to the target), which was further continuously adjusted online to ensure an overall success rate of approximately 66% in each condition.^32^ The task duration was approximately 20 minutes.

### 2.4 Behavioral analyses

Reaction times on successful trials were analyzed using a mixed‐ANOVA design, with drink (beer/water) as a within‐subject factor and group (light/at‐risk/dependent drinkers) as a between‐subject factor. Liking of the beer and water was assessed at the end of the fMRI session using Likert‐scales ranging from 1 to 10 with the questions “How much did you like the beer/water?” Liking ratings were analyzed using the same ANOVA design as for reaction times.

### 2.5 fMRI data acquisition

Imaging was conducted on a PRISMA (Fit) 3T Siemens scanner, using a 32‐channel head coil. Blood oxygen level‐dependent (BOLD) sensitive functional images were acquired with a whole brain T2*‐weighted sequence using multi‐echo echoplanar imaging (EPI) (35 axial slices, matrix 64 × 64, voxel size = 3.5 × 3.5 × 3.0 mm, repetition time = 2250 ms, echo times = [9.4, 18.8, 28.2, 37.6 ms], flip angle = 90°). The BOLD data acquisition sequence was updated during the course of the study, because of the discovery of MRI noise artefacts. The sequence parameters remained identical, except for the slice order, which changed from ascending to interleaved. We took some measures in our analyses to (a) remove the artefacts and (b) model the change in scanning sequence halfway through the study (see below). In addition, we matched across the three groups the proportion of participants scanned with the initial versus updated sequence (60% versus 40%, respectively). A high‐resolution T1 scan was acquired in each participant (192 sagittal slices, field of view 256 mm, voxel size = 1.0 × 1.0 × 1.0 mm, repetition time = 300 ms, echo time 3.03 ms).

### 2.6 fMRI data analyses

Preprocessing steps were conducted in SPM8 (http://www.fil.ion.ucl.ac.uk/spm). For each volume, the four echo images were combined into a single one, weighing all echoes equally. Standard preprocessing steps were performed on the functional data: realignment to the first image of the time series, coregistration to the structural image, normalization to MNI space based on the segmentation and normalization of the structural image, and spatial smoothing with an 8‐mm Gaussian kernel. In addition, two cleaning methods were incorporated into the pipeline to ensure optimal removal of artefacts and thorough denoising of the data: (a) a principal component analyses (PCA) to filter out slice‐specific noise components^33^ before preprocessing and (b) an independent component analysis (ICA)‐based automatic removal of motion artifacts using FMRIB Software Library (FSL) (http://www.fmrib.ox.ac.uk/fsl) after preprocessing (ICA‐AROMA).^34^ This pipeline has previously been found to be efficient to take care of the MRI noise artefacts identified in the first half of our data.^35^

After preprocessing, the data were modelled using a general linear model. The anticipation phase was modelled with a boxcar function as the combination of the cue and delay periods (duration 3.5‐9.5 seconds). The outcome notification phase was modelled with separate regressors for correct and incorrect responses using stick‐functions. The delivery phase was modelled with a boxcar function as the combination of the drink and swallow periods for correct trials (duration 7 seconds). The beer and water conditions were modelled separately. Six motion parameters were included, and a temporal high‐pass filter with a cutoff of 128 seconds was applied to remove low‐frequency noise. For each task phase (anticipation, outcome notification, and delivery), contrast images were calculated for beer>water and then entered in second‐level analyses.

In order to validate the task, we first examined the brain activity elicited by the beer>water contrast across all participants using whole‐brain one‐sample *t* tests, separately for each task phase. We used the same procedure to further examine the brain activity elicited by each drink separately, using first‐level beta images for the beer or water condition.

To examine group differences, we performed separate whole‐brain one‐way ANOVAs for each task phase, with group as a between‐subject factor (light/at‐risk/dependent drinkers). Additionally, we performed a whole‐brain regression analysis across all participants to identify brain regions in which activity elicited by the beer>water contrast would scale with a continuous measure of drinking level. For this, we computed a continuous measure reflecting the common variance across the AUDIT and weekly drinking scores. Specifically, we used a PCA (using “PCA” in MATLAB) that reduced the correlation between these scores while retaining most of their information. We selected the first principal component as a composite measure of drinking level that explained 96.0% of the common variance across the AUDIT and weekly drinking scores. The scanning sequence (before/after discovery of artefacts) was added as a binary covariate of no interest in all fMRI analyses. All T‐maps were thresholded with a voxel‐level uncorrected *P* < .001, combined with a cluster‐level family‐wise error (FWE) corrected *P* < .05, accounting for multiple comparisons across the whole brain. The F‐maps assessing group differences were thresholded with a voxel‐level FWE corrected *P* < .05 across the whole brain (since cluster‐level correction is not available for F‐maps in SPM8).

Given our a priori hypothesis about the VS, region‐of‐interest (ROI) analyses were performed using an anatomical mask of the VS.^36^ Percent signal change for the beer and water conditions was extracted in this ROI using the rfxplot toolbox^37^ and analyzed using frequentist ANOVAs in SPSS, as well as by Bayesian statistics using JASP.^38^

## 3 RESULTS

### 3.1 Behavioral results

The average success rate of 65% (SD: ± 6) approached the intended 66%. Average reaction times are shown in Table 2. Results showed neither a significant main effect of group (*F*_(2,147)_ = .345, *P =* .709) or drink (*F*_(1,147)_ = .300, *P =* .585), nor a group*drink interaction (*F*_(2,147)_ = 1.213, *P =* .300). These results suggest that reaction times, a proxy for motivation in this task, were comparable across drinks and groups. Liking ratings are shown in Table 2 (ratings were available for 136 participants because we only included these ratings after the 14th participant). Results revealed a main effect of drink (*F*_(1,133)_ = 35.302, *P* < .001), with higher liking ratings for water, compared with beer. No main effect of Group (*F*_(2,133)_ = .322, *P =* .726) or group*drink interaction (*F*_(2,133)_ = .335, *P =* .716) was observed. These results suggest that participants across the three groups liked the water more than the beer in the BID task.

**Table 2:** Behavioral results for the beer incentive delay task

	Light Drinkers (n = 39)	At‐risk Drinkers (n = 64)	Dependent Drinkers (n = 47)	Statistics
Reaction time beer (ms)	338 ± 55	332 ± 54	325 ± 45	*F*_(2,147)_ = 1.858, *P =* .300
Reaction time water (ms)	333 ± 43	335 ± 39	333 ± 45	
Liking beer (1‐10)	5.73 ± 1.7	5.80 ± 1.7	5.91 ± 1.8	*F*_(2,133)_ = .335, *P =* .716
Liking water (1‐10)	6.82 ± 1.4	7.13 ± 1.3	6.89 ± 1.1	

*Note*. Mean ± SD. Statistics reported for the group*drink interaction effects.

[Correction added on 22 May after first online publication: Data for average reaction times in Table 2 have been updated in this version]

### 3.2 Imaging results

**Figure 2:**
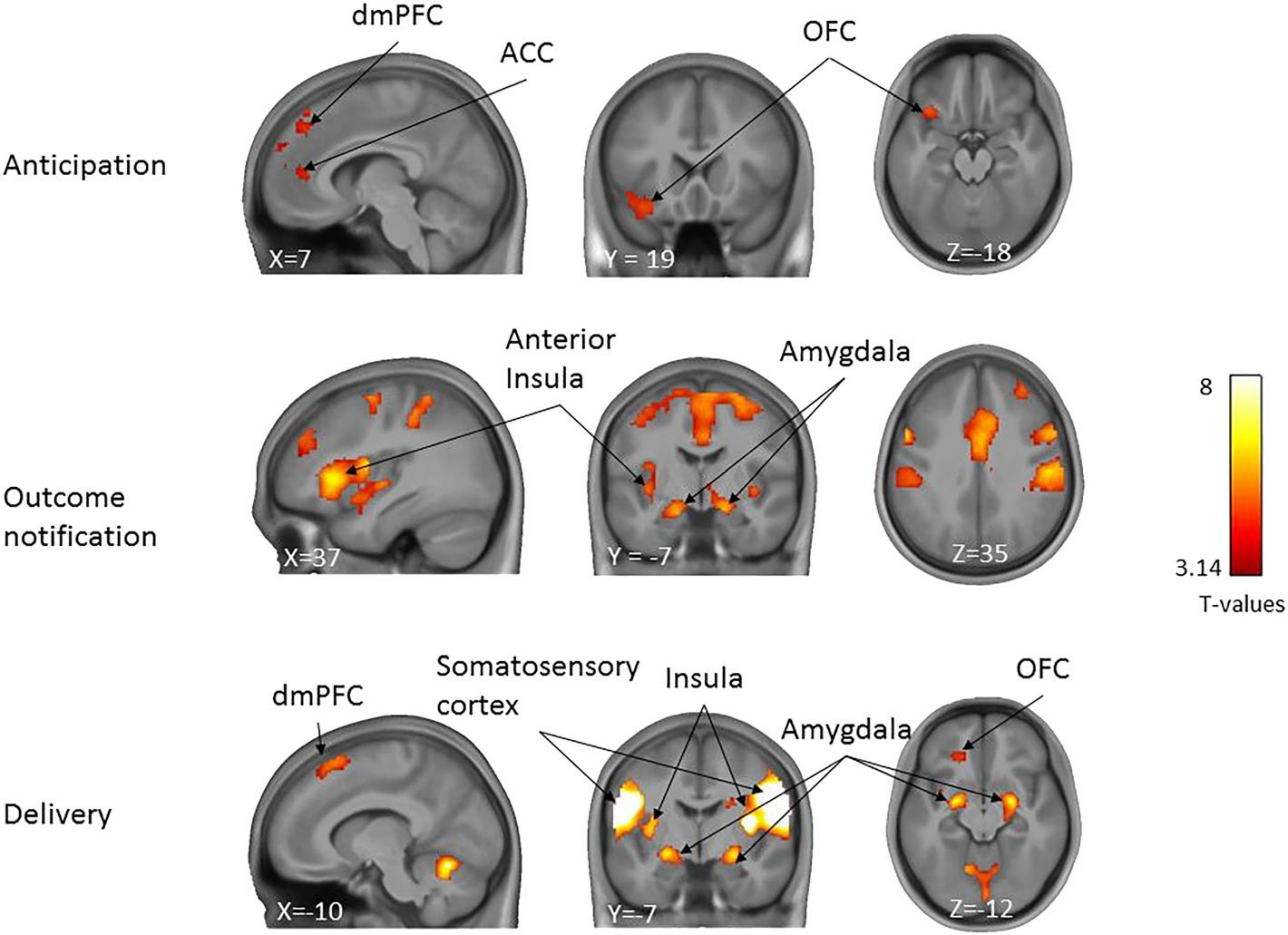
Whole brain responses for the contrasts beer>water for the anticipation, outcome notification, and delivery phases of the task, across all participants (n = 150). T‐maps are overlaid on an average anatomical scan of all participants (display threshold: voxel‐level uncorrected *P* < .001, combined with cluster‐level family‐wise error (FWE) corrected *P* < .05)

All analyses referred to in this section can be accessed at https://neurovault.org/collections/TOHDTVIQ. First, we examined brain responses to beer compared with water, in the three different phases of the task and across all participants (Figure 2). These analyses revealed that during the anticipation phase, the right dorsal medial PFC (dmPFC) [x, y, z = 7, 40, 40, *T* = 4.41], the left orbital frontal cortex (OFC) [x, y, z = –36, 23, –18, *T* = 4.94] and the anterior cingulate cortex (ACC) [x, y, z = 0, 43, 18, *T* = 4.21] responded more strongly to the anticipation of beer compared with water. During the outcome notification phase, the right anterior insula [x, y, z = 37, 23, 2, *T* = 7.83] and bilateral amygdala [x, y, z = –18, –4, –18, *T* = 6.56; 20, –4, 15, *T* = 6.73] showed increased activity upon the notification of a beer compared with water. Finally, during the delivery phase, when individuals tasted the beer compared with water, stronger activations were found in the bilateral somatosensory cortex [x, y, z = 60, –4, 25, *T* = 11.43; –53, –7, 25, *T* = 11.41], bilateral amygdala [x, y, z = –23, –4, –12, *T* = 8.07; 24, –4, –12, *T* = 8.07], bilateral insula [x, y, z = 34, –7, 12, *T* = 10.78; –33, –10, 12, *T* = 8.10], dmPFC [x, y, z = –10, 23, 60, *T* = 5.43] and left OFC [x, y, z = ‐20, 33, –10, *T* = 5.00]. Please see Table S2 for a complete list of activation foci for all task phases.

**Figure 3:**
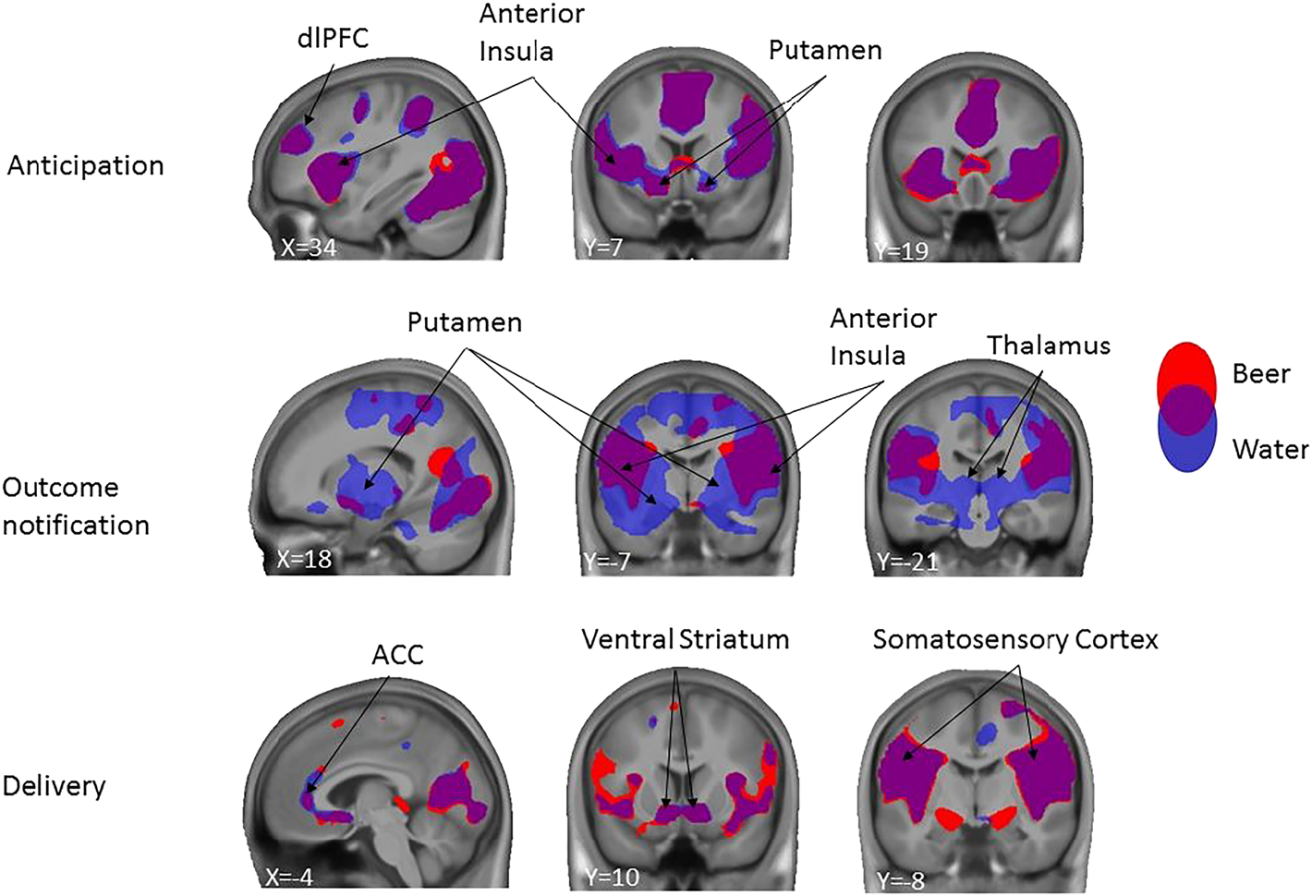
Whole brain responses to beer (blue) and water (red) for the anticipation, outcome notification, and delivery phases of the task, across all participants (n = 150). Purple areas are active in both conditions. Binarized T‐maps are overlaid on an average anatomical scan of all participants (display threshold: voxel‐level uncorrected *P* < .001, combined with cluster‐level family‐wise error (FWE) corrected *P* < .05)

The beer versus water contrast did not elicit the expected reward‐related activations in the striatum. In addition, liking ratings revealed that water was rated as more pleasurable than beer. We thus reasoned that the lack of striatal activity in the beer>water contrast might reflect the fact that the water condition elicits comparable or even higher striatal activity compared with the beer condition. To test this hypothesis, we examined brain activation patterns for the beer and water conditions separately. We found that across the three phases of the task, the beer and water conditions indeed recruit very similar brain regions including the VS and putamen, insula, dorso lateral PFC, ACC, and somatosensory cortex (Figure 3, for other foci, see Table S3). This observation confirms that the reward‐related brain network is activated in this task, but to a similar extent in the beer and water conditions, thereby explaining why their direct contrast does not produce the expected activation in the striatum.

Then, we examined whether the groups differed in their brain responses to the beer versus water conditions in any of the three phases of the task. In contrast to our hypothesis, we did not observe any significant differences surviving multiple comparisons across the whole brain between light drinkers, at‐risk drinkers, and dependent drinkers.

In order to further examine individual differences, we performed a regression analysis using our composite measure of drinking level (see Methods) as a regressor, across all participants. In line with the results of the above group analysis, this regression analysis did not reveal any brain activity in the beer>water contrast scaling with drinking level, in any of the three phases of the task.

**Figure 4:**
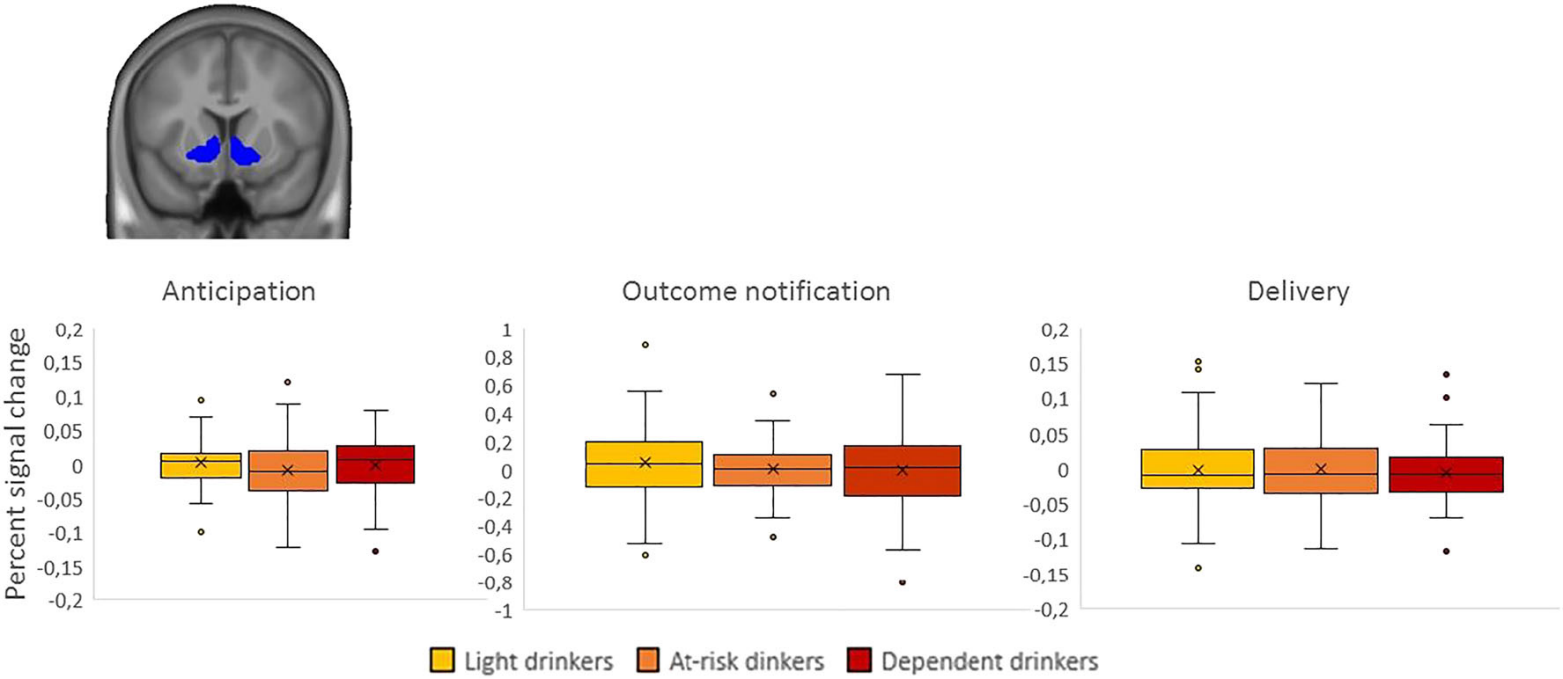
Percent signal change in the ventral striatal region‐of‐interest (ROI) for the contrast beer>water. Box height represents the interquartile range (IQR), black lines represent the median, crosses represent the mean, and whiskers represent the largest and smallest values no further than 1.5*IQR. Single data points are values located outside the whiskers. Frequentists statistics show no significant differences between groups, while Bayesian statistics provide moderate to strong evidence in favor of no group differences. Note: The scales are different between the figures because of differences in the amplitude of the blood oxygen level‐dependent (BOLD) response for the various phases of the task

Finally, we performed an ROI analysis restricted to an anatomical mask of the VS. Specifically, we extracted the percent signal change for the beer and water conditions for the various phases of the task and examined potential group differences for the beer>water contrast using a one‐way ANOVA (Figure 4). Again, the results showed no group differences during the anticipation (*F*_(2,147)_ = .955, *P =* .387), outcome notification (*F*_(2,147)_ = .511, *P =* .601), and delivery phases (*F*_(2,147)_ = .097, *P =* .908). In order to assess whether this lack of significant group differences can be interpreted as evidence for the null hypothesis (H0, no group difference), we performed Bayesian analyses in Jeffreys's Amazing Statistics Program (JASP), using a default Cauchy prior with a scale parameter of 0.5.^38^ The Bayes factor quantifying the relative evidence in favor of H0 over H1 (significant difference between groups) for the anticipation phase was BF01 = 6.557, thus providing moderate evidence for the null hypothesis of no group difference. Similarly, the Bayes factors for the outcome notification and delivery phases were BF01 = 9.710 and BF01 = 13.679, respectively, indicating strong evidence for the null hypothesis of no group difference in terms of VS activation. Sensitivity analyses controlling for smoking status did not qualitatively affect the results of the whole brain or ROI analyses.

## 4 DISCUSSION

Across groups, our results revealed increased brain activity in reward‐related brain areas during the anticipation, outcome notification, and delivery of beer compared with water, suggesting that our novel task design is well‐suited to examine the processing of alcohol‐related rewards. Yet, in contrast to our hypotheses, no brain activity was found in the VS in the beer vs water comparison, and no group differences were observed between light, at‐risk, and dependent drinkers in any of the phases of the task. While disrupted reward processing has been implicated as a core component of substance use disorders, brain responses to anticipating and receiving beer did not differentiate drinkers with different levels of alcohol use in our study. If replicated, our findings would suggest that individual differences in alcohol use may not be associated with critical differences in the processing of alcohol‐related rewards in the brain.

For validation purposes, we first examined whether the contrast of beer vs water elicited the expected activations in reward‐related brain areas. Across the various phases of the task, we found such activations in the dmPFC, ACC, OFC, and amygdala, which are commonly activated by reward or salient stimuli, in the particular in the context of gustatory stimulation.^10^, ^39^, ^40^ More specifically, during the anticipation phase, we found higher activity for the beer vs water condition in the mPFC, ACC, and OFC, areas known to be involved in the perception of craving‐related stimuli^41^ and the computation of anticipated reward value.^42^ During the outcome notification phase, we found higher activity in the insula and amygdala, both associated with the evaluation of the affective properties of stimuli.^17^, ^20^, ^43^, ^44^ Finally, during the beer delivery phase, we found higher activity in the somatosensory cortex, amygdala and insula, areas that are known to play a role in the evaluation of experienced reward and feedback, in particular in the context of food rewards.^10^, ^45^

In contrast to our hypothesis, we did not find activation differences between the beer and water conditions in the VS. Further analyses revealed that this was at least partly driven by the beer and water conditions activating the VS to a similar extent. Unexpectedly, we also found that reaction times (ie, motivation) following beer‐ and water‐predicting cues were similar, and that liking ratings for the beer condition were a bit lower than for the water condition. Presumably, drinking small sips of beer of water through a tube feels very different and less pleasurable than drinking from a glass, and water might also become rewarding under these circumstances. Interestingly, our results are in line with previous reports showing that water is similar to other caloric beverages in terms of subjective liking and wanting,^46^ and activates a large brain network including reward‐related regions.^47^ These observations call for the use of artificial saliva as a more neutral control condition,^48^ as well as a better evaluation of the hedonic properties of beer delivered through a tube in future fMRI studies. Importantly though, the lack of VS activity when comparing the beer and water conditions across all participants does not prevent us from examining group differences in this area, as it could be that VS activity is only present in individuals with relatively high drinking levels.

However, in contrast with this hypothesis, we found no differences between light, at‐risk, and dependent drinkers when examining whole brain responses to beer vs water, in any of the phases of the BID task. In the VS, Bayesian statistics further provided moderate to strong evidence in favor of the null hypothesis of no group difference. While these findings appear to be at odds with major reward‐related addiction theories,^49^, ^50^ our results are in line with a previous meta‐analysis showing no differences in brain responses to visual alcohol cues between dependent and nondependent drinkers.^11^ Our results also concur with those of a recent study that used an alcohol‐delivery protocol in the scanner and showed no reward‐related brain activity following the unpredictable delivery of alcohol vs water, as well as no reward‐related differences between alcohol‐dependent individuals and social drinkers.^21^ Below, we propose several explanations for the lack of group differences in our study.

First, our participants were relatively young (M_age_ = 21.98), and none of them was seeking treatment for their alcohol use, which means that our dependent group might represent a mild form of AUD. Moreover, at this age, the cumulative exposure to alcohol is still limited both in time and quantity, compared with older AUD populations. These specificities might play a role in the absence of reward‐related brain abnormalities in our study, which might only arise in older, treatment‐seeking AUD populations. If so, that would suggest that such abnormalities are a consequence of alcohol use rather than a predisposing factor. In support of this interpretation, a review of the literature indicates that most studies reporting positive correlations between level of use and reward‐related brain activation included dependent individuals who were in treatment.^4^ Another feature of our study that could explain the lack of group differences is the use of abstract cues in the task. Indeed, it has been suggested that sensitized brain responses to incentive cues in addiction would only be observed when using explicit addiction‐related cues, such as alcohol pictures.^9^ In contrast, abstract and nonfamiliar cues like geometric shapes might lead to blunted reward‐related brain responses. Future studies should directly test this prediction.

Alternatively, it may be that these “bottom‐up” brain responses to alcohol‐related rewards simply do not differentiate different types of drinkers and that other “top‐down” factors such as impaired prefrontal‐based self‐control,^51^, ^52^ diminished goal‐directed behavior,^53^, ^54^ and impaired decision making and learning^8^, ^55^ are more relevant in explaining individual differences in drinking behavior. In addition, a less often studied factor is the social aspect of alcohol use; it is known that alcohol is most often consumed in social settings and for social reasons,^56^, ^57^ with peer influences and imitation of drinking behavior acting as powerful predictors of use.^58^ For young adults such as those included in the present study, drinking alcohol may only be rewarding when it is accompanied by social interaction. Therefore, future research may further examine this social aspect of alcohol use in relation to reward processing.

While the relatively large sample size is a major strength of the current study, some limitations should be acknowledged. First, the ecological validity of our task can be questioned, as individuals were receiving sips of beer through a tube, while lying down in the MRI scanner. This is obviously a different experience than having a full drink from a glass in a more relaxing setting. Yet, to date, this is the closest way to examine brain responses to the taste of beer. Second, while all participants liked beer, it did not have to be their preferred drink, which may have reduced the magnitude of their brain responses to the taste of beer. Third, this study is cross‐sectional, and longitudinal data are needed to examine whether brain responses to beer can predict future alcohol use. Eventually, such data will provide insight into the risk and resilience factors regarding the transition from alcohol use to AUD. Finally, it is important to note that the focus of our study on male participants precludes any generalization of our findings to the female population.

To conclude, in this group of young adults, brain responses to the anticipation and consumption of beer were not related to individual differences in the level of alcohol use, thereby challenging the role of alcohol‐related reward processing in explaining AUD.

## Supporting information


**Figure S1**: Flow chart of the entire data collection
**Table S1**: Overview of all collected data in the study
**Table S2**: Whole brain activations for the comparison between beer and water in the three phases of the BID task
**Table S3**: Whole brain activations for the beer and water conditions compared with the implicit baseline in the three phases of the BID task

## References

[1] Hingson RW , Zha W , Weitzman ER . Magnitude of and trends in alcohol‐related mortality and morbidity among US college students ages 18‐24, 1998‐2005. J Stud Alcohol Drugs. 2009;16:12‐20. 10.15288/jsads.2009.s16.12 PMC2701090 19538908

[2] Rehm J , Mathers C , Popova S , Thavorncharoensap M , Teerawattananon Y , Patra J . Global burden of disease and injury and economic cost attributable to alcohol use and alcohol‐use disorders. The Lancet. 2009;373(9682):2223‐2233. 10.1016/S0140-6736(09)60746-7 19560604

[3] Luijten M , Schellekens AF , Kühn S , Machielse MWJ , Sescousse G . Disruption of reward processing in addiction: an image‐based meta‐analysis of functional magnetic resonance imaging studies. JAMA Psychiat. 2017;74(4):387‐398. 10.1001/jamapsychiatry.2016.3084 28146248

[4] Hommer DW , Bjork JM , Gilman JM . Imaging brain response to reward in addictive disorders. Ann N Y Acad Sci. 2011;1216(1):50‐61. 21272010 10.1111/j.1749-6632.2010.05898.x

[5] Galandra C , Basso G , Cappa S , Canessa N . The alcoholic brain: neural bases of impaired reward‐based decision‐making in alcohol use disorders. Neurol Sci. 2017;39:423‐435. 29188399 10.1007/s10072-017-3205-1

[6] Volkow ND , Morales M . The brain on drugs: from reward to addiction. Cell. 2015;162(4):712‐725. 26276628 10.1016/j.cell.2015.07.046

[7] Kuhn S , Gallinat J . Common biology of craving across legal and illegal drugs ‐ a quantitative meta‐analysis of cue‐reactivity brain response. Eur J Neurosci. 2011;33(7):1318‐1326. 21261758 10.1111/j.1460-9568.2010.07590.x

[8] Huys QJM , Deserno L , Obermayer K , Schlagenhauf F , Heinz A . Model‐free temporal‐difference learning and dopamine in alcohol dependence: examining concepts from theory and animals in human imaging. Biol Psychiatry: Cognitive Neuroscience Neuroimaging. 2016;1(5):401‐410. 10.1016/j.bpsc.2016.06.005 29560869

[9] Leyton M , Vezina P . Striatal ups and downs: Their roles in vulnerability to addictions in humans. Neurosci Biobehav Rev. 2013;37(9):1999‐2014. 23333263 10.1016/j.neubiorev.2013.01.018 PMC3743927

[10] Sescousse G , Caldú X , Segura B , Dreher JC . Processing of primary and secondary rewards: a quantitative meta‐analysis and review of human functional neuroimaging studies. Neurosci Biobehav Rev. 2013;37(4):681‐696. 23415703 10.1016/j.neubiorev.2013.02.002

[11] Schacht JP , Anton RF , Myrick H . Functional neuroimaging studies of alcohol cue reactivity: a quantitative meta‐analysis and systematic review. Addict Biol. 2013;18(1):121‐133. 22574861 10.1111/j.1369-1600.2012.00464.x PMC3419322

[12] Chase HW , Eickhoff SB , Laird AR , Hogarth L . The neural basis of drug stimulus processing and craving: an activation likelihood estimation meta‐analysis. Biol Psychiatry. 2011;70(8):785‐793. 21757184 10.1016/j.biopsych.2011.05.025 PMC4827617

[13] Saunders JB , Aasland OG , Babor TF , De La Fuente JR , Grant M . Development of the alcohol use disorders identification test (AUDIT): WHO collaborative project on early detection of persons with harmful alcohol consumption II. Addiction. 1993;88(6):791‐804. 8329970 10.1111/j.1360-0443.1993.tb02093.x

[14] Hoeppner BB , Stout RL , Jackson KM , Barnett NP . How good is fine‐grained Timeline Follow‐back data? Comparing 30‐day TLFB and repeated 7‐day TLFB alcohol consumption reports on the person and daily level. Addict Behav. 2010;35(12):1138‐1143. 20822852 10.1016/j.addbeh.2010.08.013 PMC2942970

[15] Korucuoglu O , Gladwin TE , Baas F , et al. Neural response to alcohol taste cues in youth: effects of the OPRM1 gene. Addict Biol. 2016;22:1562‐1575. 27594419 10.1111/adb.12440

[16] Claus ED , Ewing SWF , Filbey FM , Sabbineni A , Hutchison KE . Identifying neurobiological phenotypes associated with alcohol use disorder severity. Neuropsychopharmacology. 2011;36(10):2086‐2096. 21677649 10.1038/npp.2011.99 PMC3158325

[17] Filbey FM , Claus E , Audette AR , et al. Exposure to the taste of alcohol elicits activation of the mesocorticolimbic neurocircuitry. Neuropsychopharmacology. 2008;33(6):1391‐1401. 17653109 10.1038/sj.npp.1301513 PMC2856647

[18] Oberlin BG , Dzemidzic M , Tran SM , et al. Beer flavor provokes striatal dopamine release in male drinkers: mediation by family history of alcoholism. Neuropsychopharmacology. 2013;38(9):1617‐1624. 23588036 10.1038/npp.2013.91 PMC3717546

[19] Oberlin BG , Dzemidzic M , Harezlak J , et al. Corticostriatal and dopaminergic response to beer flavor with both fMRI and [(11) c]raclopride positron emission tomography. Alcohol Clin Exp Res. 2016;40(9):1865‐1873. 27459715 10.1111/acer.13158 PMC5008996

[20] Filbey FM , Ray L , Smolen A , Claus ED , Audette A , Hutchison KE . Differential neural response to alcohol priming and alcohol taste cues is associated with DRD4 VNTR and OPRM1 genotypes. Alcohol Clin Exp Res. 2008;32(7):1113‐1123. 18540916 10.1111/j.1530-0277.2008.00692.x PMC2856650

[21] Cservenka A , Courtney KE , Ghahremani DG , Hutchison KE , Ray LA . Development, initial testing and challenges of an ecologically valid reward prediction error FMRI task for alcoholism. Alcohol Alcohol. 2017;52:617‐624. 28633363 10.1093/alcalc/agx037 PMC5860006

[22] Oberlin BG , Albrecht DS , Herring CM , et al. Monetary discounting and ventral striatal dopamine receptor availability in nontreatment‐seeking alcoholics and social drinkers. Psychopharmacology (Berl). 2015;232(12):2207‐2216. 25563235 10.1007/s00213-014-3850-5 PMC4545519

[23] Delgado MR , Miller MM , Inati S , Phelps EA . An fMRI study of reward‐related probability learning. Neuroimage. 2005;24(3):862‐873. 15652321 10.1016/j.neuroimage.2004.10.002

[24] Zink CF , Pagnoni G , Martin‐Skurski ME , Chappelow JC , Berns GS . Human striatal responses to monetary reward depend on saliency. Neuron. 2004;42(3):509‐517. 15134646 10.1016/s0896-6273(04)00183-7

[25] Elliott R , Newman JL , Longe OA , William Deakin JF . Instrumental responding for rewards is associated with enhanced neuronal response in subcortical reward systems. Neuroimage. 2004;21(3):984‐990. 15006665 10.1016/j.neuroimage.2003.10.010

[26] Jacobs EH , Smit AB , de Vries TJ , Schoffelmeer ANM . Neuroadaptive effects of active versus passive drug administration in addiction research. Trends Pharmacol Sci. 2003;24(11):566‐573. 14607079 10.1016/j.tips.2003.09.006

[27] Knutson B , Greer SM . Anticipatory affect: neural correlates and consequences for choice. Philos Trans R Soc Lond B Biol Sci. 2008;363(1511):3771‐3786. 18829428 10.1098/rstb.2008.0155 PMC2607363

[28] Babor TF , Higgins‐Biddle JC , Saunders JB , Monteiro MG . AUDIT: the alcohol use disorders identification test: guidelines for use in primary health care. Geneva: World Health Organization; 2001.

[29] Ihssen N , Cox WM , Wiggett A , Fadardi JS , Linden DEJ . Differentiating heavy from light drinkers by neural responses to visual alcohol cues and other motivational stimuli. Cereb Cortex. 2011;21(6):1408‐1415. 21045002 10.1093/cercor/bhq220

[30] Monshouwer K , Tuithof M , van Dorsselaer S . Factsheet riskant alcoholgebruik in Nederland [Factsheet risky alcohol use in the Netherlands]. https://www.trimbos.nl/docs/bd1d5260-16d8-4d3f-a151-09f983d61d4c.pdf, 2018.

[31] Sheehan DV , Lecrubier Y , Sheehan KH , et al. The validity of the Mini International Neuropsychiatric Interview (MINI) according to the SCID‐P and its reliability. Eur Psychiatry. 1997;12(5):224‐231.

[32] Knutson B , Westdorp A , Kaiser E , Hommer D . FMRI visualization of brain activity during a monetary incentive delay task. Neuroimage. 2000;12(1):20‐27. 10875899 10.1006/nimg.2000.0593

[33] Viviani R , Gron G , Spitzer M . Functional principal component analysis of fMRI data. Hum Brain Mapp. 2005;24(2):109‐129. 15468155 10.1002/hbm.20074 PMC6871761

[34] Pruim RH , Mennes M , van Rooij D , Llera A , Buitelaar JK , Beckmann CF . ICA‐AROMA: A robust ICA‐based strategy for removing motion artifacts from fMRI data. Neuroimage. 2015;112:267‐277. 25770991 10.1016/j.neuroimage.2015.02.064

[35] Nieuwhof F , Bloem BR , Reelick MF , et al. Impaired dual tasking in Parkinson's disease is associated with reduced focusing of cortico‐striatal activity. Brain. 2017;140(5):1384‐1398. 28335024 10.1093/brain/awx042

[36] Murray GK , Corlett PR , Clark L , et al. Substantia nigra/ventral tegmental reward prediction error disruption in psychosis. Mol Psychiatry. 2008;13(3):267‐276. 10.1038/sj.mp.4002058 PMC2564111 17684497

[37] Glascher J . Visualization of group inference data in functional neuroimaging. Neuroinformatics. 2009;7(1):73‐82. 19140033 10.1007/s12021-008-9042-x

[38] Wagenmakers EJ , Love J , Marsman M , et al. Bayesian inference for psychology. Part II: Example applications with JASP. Psychon Bull Rev. 2017;25:58‐76. 10.3758/s13423-017-1323-7 PMC5862926 28685272

[39] Bartra O , McGuire JT , Kable JW . The valuation system: a coordinate‐based meta‐analysis of BOLD fMRI experiments examining neural correlates of subjective value. Neuroimage. 2013;76:412‐427. 23507394 10.1016/j.neuroimage.2013.02.063 PMC3756836

[40] Veldhuizen MG , Albrecht J , Zelano C , Boesveldt S , Breslin P , Lundström JN . Identification of human gustatory cortex by activation likelihood estimation. Hum Brain Mapp. 2011;32(12):2256‐2266. 21305668 10.1002/hbm.21188 PMC3123671

[41] Diekhof EK , Falkai P , Gruber O . Functional neuroimaging of reward processing and decision‐making: a review of aberrant motivational and affective processing in addiction and mood disorders. Brain Res Rev. 2008;59(1):164‐184. 18675846 10.1016/j.brainresrev.2008.07.004

[42] Haber SN , Knutson B . The reward circuit: linking primate anatomy and human imaging. Neuropsychopharmacology. 2010;35(1):4‐26. 19812543 10.1038/npp.2009.129 PMC3055449

[43] Etkin A , Buchel C , Gross JJ . The neural bases of emotion regulation. Nat Rev Neurosci. 2015;16:603‐700. 10.1038/nrn4044 26481098

[44] Ochsner KN , Silvers JA , Buhle JT . Functional imaging studies of emotion regulation: a synthetic review and evolving model of the cognitive control of emotion. Ann N Y Acad Sci. 2012;1251(1):E1‐E24. 23025352 10.1111/j.1749-6632.2012.06751.x PMC4133790

[45] Schultz W . Dopamine reward prediction error coding. Dialogues Clin Neurosci. 2016;18(1):23‐32. 27069377 10.31887/DCNS.2016.18.1/wschultz PMC4826767

[46] Wegman J , van Loon I , Smeets PAM , Cools R , Aarts E . Top‐down expectation effects of food labels on motivation. Neuroimage. 2018;173:13‐24. 29438844 10.1016/j.neuroimage.2018.02.011

[47] de Araujo IET , Kringelbach ML , Rolls ET , McGlone F . Human cortical responses to water in the mouth, and the effects of thirst. J Neurophysiol. 2003;90(3):1865‐1876. 12773496 10.1152/jn.00297.2003

[48] Veldhuizen MG , Bender G , Constable RT , Small DM . Trying to detect taste in a tasteless solution: modulation of early gustatory cortex by attention to taste. Chem Senses. 2007;32(6):569‐581. 17495173 10.1093/chemse/bjm025

[49] Robinson TE , Berridge KC . Incentive‐sensitization and addiction. Addiction. 2001;96(1):103‐114. 11177523 10.1046/j.1360-0443.2001.9611038.x

[50] Bjork JM , Smith AR , Chen G , Hommer DW . Mesolimbic recruitment by nondrug rewards in detoxified alcoholics: effort anticipation, reward anticipation, and reward delivery. Hum Brain Mapp. 2012;33(9):2174‐2188. 22281932 10.1002/hbm.21351 PMC3342463

[51] Luijten M , Machielsen MW , Veltman DJ , Hester R , de Haan L , Franken IH . Systematic review of ERP and fMRI studies investigating inhibitory control and error processing in people with substance dependence and behavioural addictions. J Psychiatry Neurosci. 2014;39(3):149‐169. 24359877 10.1503/jpn.130052 PMC3997601

[52] Tang YY , Posner MI , Rothbart MK , Volkow ND . Circuitry of self‐control and its role in reducing addiction. Trends Cogn Sci. 2015;19(8):439‐444. 26235449 10.1016/j.tics.2015.06.007

[53] Reiter AM , Deserno L , Wilbertz T , Heinze HJ , Schlagenhauf F . Risk factors for addiction and their association with model‐based behavioral control. Front Behav Neurosci. 2016;10:26. 27013998 10.3389/fnbeh.2016.00026 PMC4794491

[54] Sebold M , Nebe S , Garbusow M , et al. When habits are dangerous: alcohol expectancies and habitual decision making predict relapse in alcohol dependence. Biol Psychiatry. 2017;82(11):847‐856. 28673442 10.1016/j.biopsych.2017.04.019

[55] Reiter AM , Deserno L , Kallert T , Heinze HJ , Heinz A , Schlagenhauf F . Behavioral and neural signatures of reduced updating of alternative options in alcohol‐dependent patients during flexible decision‐making. J Neurosci. 2016;36(43):10935‐10948. 27798176 10.1523/JNEUROSCI.4322-15.2016 PMC6705653

[56] Dallas R , Field M , Jones A , Christiansen P , Rose A , Robinson E . Influenced but unaware: social influence on alcohol drinking among social acquaintances. Alcohol Clin Exp Res. 2014;38(5):1448‐1453. 24588229 10.1111/acer.12375

[57] Smit K , Groefsema M , Luijten M , Engels R , Kuntsche E . Drinking motives moderate the effect of the social environment on alcohol use: an event‐level study among young adults. J Stud Alcohol Drugs. 2015;76(6):971‐980. 26562607 10.15288/jsad.2015.76.971

[58] Larsen H , Engels RCME , Granic I , Overbeek G . An experimental study on imitation of alcohol consumption in same‐sex dyads. Alcohol Alcohol. 2009;44(3):250‐255. 19240054 10.1093/alcalc/agp002

