# Supplementary material for: Brain responses to anticipating and receiving beer: Comparing light, at‐risk, and dependent alcohol users
Source: Addict Biol. 2019 May 7;25(3):e12766. doi: 10.1111/adb.12766 (PMC7187239; doi:10.1111/adb.12766)
Supplement: Supplementary file 1 — Figure S1: Flow chart of the entire data collection Table S1: Overview of all collected data in the study Table S2: Whole brain activations for the comparison between beer and water in the three phases of the BID task Table S3: Whole brain activations for the beer and water conditions compared with the implicit baseline in the three phases of the BID task [file ADB-25-e12766-s001.docx]

Supplementary Figure 1: Flow chart of the entire data collection

**Following online screening**
n = 173

Inconsistency between self-report measures and clinical interview^1^, n = 7

**Sample started data collection**

n = 165

Dropouts during data collection, n = 6

Incorrect inclusions^2^, n = 7

Technical problems and head movement, n = 3

**Final sample submitted to data analysis**
n = 150

**Dependent drinkers**n = 47

**At-risk drinkers**
n = 64

**Light drinkers**
n = 39

*^1^The most severe group of “dependent drinkers” was defined based on the following three criteria: AUDIT score >15, more than 22 drinks/week (both verified during online screening) and DSM criteria for alcohol dependence based on MINI interview (verified during onsite screening). Thus, some participants who showed signs of problematic and heavy alcohol use during online screening (i.e. >15 on the AUDIT and more than 22 drinks/week) did not meet the criteria for alcohol dependence following the onsite screening. We decided to exclude these participants, as we deemed that this inconsistency between self-report and DSM-based measures made their categorization uncertain.*

*^2^ Incorrect inclusions did not meet the combined requirement of drinking ≤ 22 drinks/week with an AUDIT score between 0 and 15, or drinking > 22 drinks/week with an AUDIT score >15. The data from these 7 participants were discarded before performing any data analysis.*

Table S1: Overview of all collected data in the study

| When | What | How |
| --- | --- | --- |
| Screening | | |
|  | Alcohol use disorder | AUDIT |
|  | Drinks last week | TLFB |
|  | Gender | Male/female question |
|  | Age | Open question |
| MINI Interview (for the dependent group only) | | |
|  | Alcohol dependence diagnosis | MINI |
|  | Other substance use information | MINI |
| Behavioural session 1 | | |
|  | Education | Multiple choice |
|  | Age of first alcohol consumption | Open question |
|  | Frequency of drinks in the last 4 weeks | Multiple choice |
|  | Binge drinking episodes in last 4 weeks | Multiple choice |
|  | Location of alcohol consumption | Multiple choice |
|  | Drinking motives | DMQ-R |
|  | Ever drank alcohol | Yes/No question |
|  | Number of hours since last drink | Open question |
|  | Ever smoked in life | Yes/No question |
|  | Current smoker | Yes/No question |
|  | Number of hours since last cigarette | Open question |
|  | Smoking severity | FTND |
|  | Ever used drugs (sleeping pills/cannabis/cocaine/ecstacy/amphetamine/hallucinogens/opiates), if yes how many times | Open question |
|  | Impulsivity | BIS11 |
|  | State-trait Anxiety | STAI |
|  | Depression | BDI |
|  | Drinking Urge | DAQ |
|  | Imitation of alcohol use | Number of drinks consumed in Bar-lab |
|  | Confederate liking ratings | 1-9 scales |
|  | Anxiety before/during/after session | Multiple choice |
|  | Suspicion checks about study goals | Open questions |
| Behavioural session 2 | | |
|  | Number of hours since last drink | Open question |
|  | Number of hours since last cigarette | Open question |
|  | Delay Discounting | Delay discounting task |
|  | Drinking Urge | DAQ |
|  | Imitation of alcohol use | Number of drinks consumed in Bar-lab |
|  | Confederate liking ratings | 1-9 scales |
|  | Anxiety before/during/after session | Multiple choice |
|  | Suspicion checks about study goals | Open questions |
|  |  |  |
|  |  |  |
|  |  |  |
|  |  |  |
|  |  |  |
|  |  |  |
|  |  |  |

Table S1: Overview of all collected data in the study (continued)

| fMRI session | | |
| --- | --- | --- |
| When | **WHAT** | **HOW** |
|  | Number of hours since last drink | Open question |
|  | Number of hours since last cigarette | Open question |
|  | Height/Weight | Open question |
|  | Drinking Urge | DAQ |
|  | Drinking Self-Efficacy | DRSEQ |
|  | Brain responses to social alcohol cues | Social Alcohol cue Reactivity task |
|  | Brain responses to anticipating and receiving beer | Beer Incentive Delay task (current paper) |
|  | Approach/Avoidance of (social) alcohol pictures | Stimulus-Response Compatibility task |
| Follow up Baseline | |  |
|  | Alcohol use disorder | AUDIT |
|  | Average number of drinks for each day of the week | Weekly drinking |
|  | Drinking motives | DMQ-R |
| Follow up with ecological momentary assessment (14 days) | | |
|  | Number of (non)alcohol units consumed day before | Open question |
|  | Location of (non)alcohol consumption day before | Multiple choice |
|  | People with whom (non)alcohol was consumed with day before | Multiple choice |

*Table S2: Whole brain activations for the comparison between beer and water in the three phases of the BID task*

| Brain area | Hemisphere | Cluster size | MNI coordinates peak voxel | | | T-value | p-value |
| --- | --- | --- | --- | --- | --- | --- | --- |
|  |  |  | x | y | Z |  |  |
| **Anticipation phase** |  |  |  |  |  |  |  |
| Beer>Water |  |  |  |  |  |  |  |
| **Posterior Orbital Gyrus** | **L** | **207** | **-38** | **20** | **-18** | **5.27** | **0.000** |
| Posterior Orbital Gyrus | L |  | -30 | 23 | -15 | 4.67 |  |
| Inferior Frontal Gyrus | L |  | -46 | 26 | -8 | 4.08 |  |
|  |  |  |  |  |  |  |  |
| **Superior Frontal Gyrus** | **R** | **155** | **7** | **40** | **55** | **4.51** | **0.000** |
| Superior Frontal Gyrus | R |  | 4 | 43 | 42 | 4.13 |  |
| Superior Frontal Gyrus | L |  | -3 | 38 | 50 | 4.03 |  |
|  |  |  |  |  |  |  |  |
| **Anterior Cingulate Gyrus** | **L** | **460** | **-3** | **43** | **15** | **4.47** | **0.000** |
| Anterior Cingulate Gyrus | L |  | -16 | 46 | 10 | 4.22 |  |
| Anterior Cingulate Gyrus | L |  | -8 | 48 | 25 | 4.13 |  |
| Water>Beer |  |  |  |  |  |  |  |
| **Insula** | **R** | **3986** | **34** | **-7** | **10** | **8.55** | **0.000** |
| Postcentral Gyrus | R |  | 37 | -10 | 18 | 8.30 |  |
| Postcentral Gyrus | R |  | 50 | -10 | 25 | 7.36 |  |
|  |  |  |  |  |  |  |  |
| **Insula** | **L** | **4976** | **-36** | **-10** | **18** | **8.06** | **0.000** |
| Postcentral Gyrus | L |  | -46 | -14 | 20 | 7.83 |  |
| Insula | L |  | -38 | -4 | 2 | 7.68 |  |
|  |  |  |  |  |  |  |  |
| **Occipital Lobe** | **L** | **550** | **-20** | **-92** | **-12** | **5.36** | **0.000** |
| Occipital Lobe | L |  | -26 | -87 | -20 | 5.14 |  |
| Occipital Lobe | L |  | -28 | -82 | -12 | 5.06 |  |
|  |  |  |  |  |  |  |  |
| **Cerebellum** | **L** | **72** | **-20** | **-40** | **-28** | **4.78** | **0.046** |
| Cerebellum | L |  | -28 | -42 | -30 | 4.15 |  |
| Cerebellum | L |  | -23 | -50 | -30 | 4.00 |  |

*Table S2: Whole brain activations for the comparison between beer and water in the three phases of the BID task (continued)*

|  |  |  |  |  |  |  |  |
| --- | --- | --- | --- | --- | --- | --- | --- |
| **Occipital Lobe** | **R** | **284** | **37** | **-64** | **-12** | **4.48** | **0.000** |
| Occipital Lobe | R |  | 30 | -77 | -12 | 4.23 |  |
| Occipital Lobe | R |  | 32 | -90 | -8 | 4.14 |  |
|  |  |  |  |  |  |  |  |
| **Cerebellum** | **R** | **80** | **27** | **-50** | **-28** | **4.26** | **0.031** |
| Cerebellum | R |  | 34 | -47 | -30 | 3.66 |  |
|  |  |  |  |  |  |  |  |
| **Superior Parietal Gyrus** | **L** | **71** | **-28** | **-47** | **35** | **4.23** | **0.048** |
| Superior Parietal Gyrus | L |  | -23 | -64 | 38 | 3.92 |  |
| Superior Parietal Gyrus | L |  | -28 | -52 | 42 | 3.41 |  |
| **Outcome notification phase** |  |  |  |  |  |  |  |
| Beer>Water |  |  |  |  |  |  |  |
| **Precentral Gyrus** | **R** | **6742** | **54** | **10** | **28** | **9.45** | **0.000** |
| Inferior Frontal Gyrus | R |  | 57 | 10 | 15 | 8.00 |  |
| Inferior Frontal Gyrus | R |  | 40 | 26 | 5 | 7.60 |  |
|  |  |  |  |  |  |  |  |
| **Inferiolateral Parietal Lobe** | **R** | **1585** | **54** | **-20** | **40** | **7.56** | **0.000** |
| Inferiolateral Parietal Lobe | R |  | 50 | -27 | 40 | 7.46 |  |
| Inferiolateral Parietal Lobe | R |  | 57 | -20 | 28 | 6.91 |  |
| Posterior Temporal Lobe | L | 112 | -46 | -60 | 0 | 4.93 | 0.004 |
| Inferiolateral Parietal Lobe | L |  | -63 | -57 | 0 | 3.59 |  |
|  |  |  |  |  |  |  |  |
| **Superior Parietal Gyrus** | **L** | **98** | **-20** | **-52** | **60** | **4.85** | **0.008** |
| Superior Parietal Gyrus | L |  | -13 | -50 | 62 | 4.29 |  |
| Superior Parietal Gyrus | L |  | -13 | -62 | 58 | 3.22 |  |
|  |  |  |  |  |  |  |  |
| **Middle Frontal Gyrus** | **R** | **153** | **34** | **43** | **32** | **4.66** | **0.001** |
|  |  |  |  |  |  |  |  |
| **Inferiolateral Parietal Lobe** | **L** | **275** | **-53** | **-27** | **38** | **4.55** | **0.000** |
| Inferiolateral Parietal Lobe |  |  | -60 | -27 | 32 | 4.31 |  |

*Table S2: Whole brain activations for the comparison between beer and water in the three phases of the BID task (continued)*

| Inferiolateral Parietal Lobe |  |  | -50 | -20 | 25 | 3.52 |  |
| --- | --- | --- | --- | --- | --- | --- | --- |
| Water>Beer |  |  |  |  |  |  |  |
| **Inferiorlateral Parietal Lobe** | **L** | **240** | **-43** | **-60** | **40** | **4.85** | **0.000** |
| Inferiolateral Parietal Lobe | L |  | -56 | -60 | 35 | 4.62 |  |
| Inferiolateral Parietal Lobe | L |  | -48 | -54 | 32 | 3.92 |  |
|  |  |  |  |  |  |  |  |
| **Inferiorlateral Parietal Lobe** | **L** | **106** | **-26** | **-90** | **10** | **4.28** | **0.005** |
| Inferiolateral Parietal Lobe | L |  | -26 | -92 | 22 | 3.99 |  |
| Inferiolateral Parietal Lobe | L |  | -20 | -82 | 18 | 3.73 |  |
|  |  |  |  |  |  |  |  |
| **Middle Frontal Gyrus** | **L** | **94** | **-38** | **20** | **42** | **4.19** | **0.010** |
| Middle Frontal Gyrus | L |  | -30 | 13 | 42 | 3.42 |  |
|  |  |  |  |  |  |  |  |
| **Inferiorlateral Parietal Lobe** | **R** | **74** | **54** | **-54** | **35** | **4.13** | **0.028** |
| Inferiolateral Parietal Lobe | R |  | 50 | -50 | 25 | 3.80 | 0.000 |
| **Delivery phase** |  |  |  |  |  |  |  |
| Beer>Water |  |  |  |  |  |  |  |
| **Insula** | **R** | **2974** | **34** | **-7** | **10** | **12.30** | **0.000** |
| Poscentral Gyrus | R |  | 60 | -4 | 25 | 12.18 |  |
| Amygdala | R |  | 22 | -2 | -15 | 9.34 |  |
|  |  |  |  |  |  |  |  |
| **Precentral Gyrus** | **L** | **2818** | **-53** | **-7** | **25** | **12.13** | **0.000** |
| Amygdala | L |  | -23 | -4 | -12 | 9.86 |  |
| Insula | L |  | -33 | -10 | 12 | 9.32 |  |
|  |  |  |  |  |  |  |  |
| **Cerebellum** | **L** | **1290** | **-16** | **-62** | **-20** | **11.47** | **0.000** |
| Cerebellum | R |  | 17 | -62 | -20 | 9.92 |  |
| Cerebellum | R |  | 2 | -82 | -15 | 6.45 |  |
|  |  |  |  |  |  |  |  |
| **Superior Frontal Gyrus** | **L** | **304** | **-10** | **23** | **60** | **5.55** | **0.000** |

*Table S2: Whole brain activations for the comparison between beer and water in the three phases of the BID task (continued)*

|  |  |  |  |  |  |  |  |
| --- | --- | --- | --- | --- | --- | --- | --- |
| **Anterior Orbital Gyrus** | **L** | **142** | **-20** | **33** | **-10** | **5.32** | **0.002** |
|  |  |  |  |  |  |  |  |
| **Superior Frontal Gyrus** | **R** | **76** | **20** | **56** | **30** | **4.94** | **0.042** |
|  |  |  |  |  |  |  |  |
| **Superior Frontal Gyrus** | **L** | **98** | **-10** | **53** | **10** | **4.13** | **0.015** |
| Superior Frontal Gyrus |  |  | -6 | 53 | 30 | 3.86 |  |
| Superior Frontal Gyrus |  |  | -16 | 50 | 32 | 3.32 |  |
| Water>Beer |  |  |  |  |  |  |  |
| **Occipital Lobe** | **L** | **2205** | **-38** | **-84** | **-8** | **7.75** | **0.000** |
| Occipital Lobe | L |  | -26 | -82 | 28 | 7.09 |  |
| Occipital Lobe | L |  | -26 | -92 | -10 | 7.09 |  |
|  |  |  |  |  |  |  |  |
| **Occipital Lobe** | **R** | **3628** | **34** | **-70** | **25** | **7.52** | **0.000** |
| Occipital Lobe | R |  | 40 | -77 | 12 | 7.10 |  |
| Occipital Lobe | R |  | 37 | -77 | 22 | 7.08 |  |
|  |  |  |  |  |  |  |  |
| **Posterior Cingulate Gyrus** | **R** | **229** | **10** | **-20** | **32** | **5.11** | **0.000** |
| Posterior Cingulate Gyrus | L |  | -8 | -7 | 35 | 4.69 |  |
| Posterior Cingulate Gyrus | R |  | 7 | 0 | 32 | 4.67 |  |
|  |  |  |  |  |  |  |  |
| **Middle Frontal Gyrus** | **R** | **138** | **24** | **56** | **2** | **4.90** | **0.003** |
|  |  |  |  |  |  |  |  |
| **Inferior Frontal Gyrus** | **R** | **215** | **37** | **16** | **22** | **4.71** | **0.000** |
| Middle Frontal Gyrus | R |  | 40 | 8 | 38 | 4.31 |  |
| Middle Frontal Gyrus | R |  | 37 | 3 | 48 | 4.09 |  |
|  |  |  |  |  |  |  |  |

Note: Reported coordinates correspond to the three highest peak voxels more than 8 mm apart in each cluster. P-values are cluster level FWE- corrected.

*Table S3: Whole brain activations for the beer and water conditions compared with the implicit baseline in the three phases of the BID task*

| Brain area | Hemisphere | Cluster size | MNI coordinates peak voxel | | | *T-value* | *p*-value |
| --- | --- | --- | --- | --- | --- | --- | --- |
|  |  |  | x | y | z |  |  |
| **Anticipation phase** |  |  |  |  |  |  |  |
| Beer |  |  |  |  |  |  |  |
| **Occipital Lobe** | **R** | **5848** | **37** | **-87** | **2** | **20.22** | **0.000** |
| Occipital Lobe | R |  | 32 | -80 | -12 | 19.26 |  |
| Occipital Lobe | R |  | 30 | -90 | 8 | 18.90 |  |
| Occipital Lobe | R |  | 24 | -82 | -10 | 17.99 |  |
| Occipital Lobe | R |  | 20 | -84 | -8 | 17.85 |  |
| Occipital Lobe | R |  | 22 | -97 | 2 | 15.57 |  |
| Occipital Lobe | R |  | 34 | -62 | -12 | 13.61 |  |
| Parietal Lobe | R |  | 64 | -34 | 25 | 12.06 |  |
| Superior Parietal Gyrus | R |  | 30 | -47 | 45 | 9.25 |  |
| Cerebellum | R |  | 42 | -54 | -30 | 8.63 |  |
| Parietal Lobe | R |  | 60 | -20 | 25 | 7.54 |  |
| Parietal Lobe | R |  | 17 | -72 | 35 | 7.44 |  |
| Parietal Lobe | R |  | 20 | -70 | 32 | 7.04 |  |
| Superior Parietal Gyrus | R |  | 27 | -62 | 8 | 6.28 |  |
| Occipital Lobe | R |  | 22 | -42 | 20 | 6.15 |  |
| Superior Parietal Gyrus | R |  | 20 | -60 | 35 | 5.74 |  |
|  |  |  |  |  |  |  |  |
| **Superior Frontal Gyrus** | R | 16798 | 4 | 13 | 52 | 17.97 | 0.000 |
| Occipital Lobe | L |  | -26 | -82 | -12 | 17.57 |  |
| Superior Frontal Gyrus | R |  | 2 | 16 | 42 | 17.36 |  |
| Occipital Lobe | L |  | -30 | -74 | -12 | 17.04 |  |
| Inferior Frontal Gyrus | R |  | 34 | 28 | 2 | 17.03 |  |
| Inferior Frontal Gyrus | R |  | 32 | 26 | 5 | 16.93 |  |
| Insula | R |  | 40 | 18 | -2 | 16.79 |  |
| Posterior Orbital Gyrus | R |  | 32 | 23 | -10 | 16.73 |  |
|  |  |  | -28 | -92 | 5 | 16.42 |  |
| Superior Frontal Gyrus | R |  | 2 | 0 | 60 | 16.38 |  |
| Insula | L |  | -33 | 20 | -10 | 16.22 |  |

*Table S3: Whole brain activations for the beer and water conditions compared with the implicit baseline in the three phases of the BID task (continued)*

| Insula | L |  | -30 | 23 | 8 | 16.08 |  |
| --- | --- | --- | --- | --- | --- | --- | --- |
| Insula | L |  | -38 | 16 | -5 | 15.91 |  |
| Occipital Lobe | L |  | -40 | -77 | -10 | 15.91 |  |
| Occipital Lobe | L |  | -38 | -87 | 0 | 15.74 |  |
| Occipital Lobe | L |  | -36 | -92 | 2 | 15.31 |  |
|  |  |  |  |  |  |  |  |
| **Middle Frontal Gyrus** | R | 480 | 27 | 53 | 30 | 9.18 | 0.000 |
| Middle Frontal Gyrus | R |  | 30 | 48 | 28 | 8.84 |  |
|  |  |  |  |  |  |  |  |
| **Middle Frontal Gyrus** | L | 204 | -30 | 50 | 30 | 6.95 | 0.001 |
|  |  |  |  |  |  |  |  |
|  |  |  |  |  |  |  |  |
| **Cerebellum** | R | 161 | 4 | -64 | -18 | 5.67 | 0.004 |
| Cerebellum | R |  | 2 | -62 | -5 | 3.91 |  |
| Water |  |  |  |  |  |  |  |
| **Occipital Lobe** | R | 26098 | 37 | -90 | 5 | 19.49 | 0.000 |
| Occipital Lobe | R |  | 40 | -80 | -10 | 19.40 |  |
| Occipital Lobe | R |  | 32 | -87 | 5 | 19.17 |  |
| Occipital Lobe | R |  | 32 | -77 | -12 | 18.94 |  |
| Superior Frontal Gyrus | R |  | 7 | 0 | 60 | 18.91 |  |
| Occipital Lobe | L |  | -28 | -82 | -12 | 18.90 |  |
| Superior Frontal Gyrus | L |  | 4 | 0 | 55 | 18.78 |  |
| Superior Frontal Gyrus | L/R |  | 0 | -2 | 60 | 18.67 |  |
| Superior Frontal Gyrus | L |  | 4 | 13 | 52 | 18.44 |  |
| Insula | R |  | -30 | 20 | 8 | 17.94 |  |
| Insula | L |  | 32 | 23 | 2 | 17.81 |  |
| Occipital Lobe | L |  | -36 | -80 | -10 | 17.45 |  |
| Occipital Lobe | R |  | 20 | -87 | -8 | 17.44 |  |
| Occipital Lobe | L |  | -26 | -92 | 8 | 17.38 |  |
| Occipital Lobe | L |  | -40 | -77 | -10 | 17.26 |  |
| Occipital Lobe | L |  | -33 | -90 | 2 | 16.29 |  |
| **Middle Frontal Gyrus** | R | 563 | 34 | 48 | 28 | 8.73 | 0.000 |

*Table S3: Whole brain activations for the beer and water conditions compared with the implicit baseline in the three phases of the BID task (continued)*

| Middle Frontal Gyrus | R |  | 34 | 43 | 28 | 8.43 |  |
| --- | --- | --- | --- | --- | --- | --- | --- |
| Middle Frontal Gyrus | R |  | 32 | 38 | 25 | 8.34 |  |
| Middle Frontal Gyrus | R |  | 44 | 48 | 2 | 4.43 |  |
| Middle Frontal Gyrus | R |  | 47 | 50 | 0 | 4.29 |  |
|  |  |  |  |  |  |  |  |
| **Middle Frontal Gyrus** | L | 153 | -33 | 48 | 30 | 6.33 | 0.000 |
|  |  |  |  |  |  |  |  |
| **Outcome notification phase** |  |  |  |  |  |  |  |
| Beer |  |  |  |  |  |  |  |
| **Lingual Gyrus** | R | 4657 | 17 | -87 | -8 | 18.64 | 0.000 |
| Occipital Lobe | L |  | -13 | -92 | -8 | 17.35 |  |
| Lingual Gyrus | L |  | -28 | -80 | -12 | 14.67 |  |
| Cerebellum | R |  | 17 | -60 | -22 | 11.97 |  |
| Cerebellum | L |  | -16 | -60 | -22 | 11.91 |  |
| Lingual Gyrus | L |  | -6 | -67 | 8 | 8.21 |  |
| Posterior Temporal Lobe | L |  | -38 | -44 | -20 | 7.84 |  |
| Cuneus | L |  | -6 | -77 | 10 | 7.59 |  |
| Lingual Gyrus | R |  | 10 | -67 | 10 | 7.22 |  |
| Posterior Temporal Lobe | L |  | -38 | -54 | -15 | 6.36 |  |
| Superior Parietal Gyrus | R |  | 20 | -57 | 20 | 5.86 |  |
| Superior Parietal Gyrus | L |  | -13 | -60 | 20 | 5.66 |  |
| Fusiform Gyrus | L |  | -38 | -22 | -25 | 3.72 |  |
| Cuneus | L |  | -8 | -80 | 28 | 3.67 |  |
|  |  |  |  |  |  |  |  |
| **Postcentral Gyrus** | L | 3185 | -38 | -12 | 22 | 13.57 | 0.000 |
| Postcentral Gyrus | L |  | -66 | -17 | 25 | 12.48 |  |
| Postcentral Gyrus | L |  | -58 | -12 | 15 | 11.33 |  |
| Insula | L |  | -36 | -10 | 12 | 11.18 |  |
| Insula | L |  | -38 | 0 | -8 | 10.57 |  |
| Parietal Lobe | L |  | -66 | -27 | 25 | 10.38 |  |
| Parietal Lobe | L |  | -63 | -30 | 28 | 10.37 |  |
| Precentral Gyrus | L |  | -58 | -7 | 38 | 10.00 |  |

*Table S3: Whole brain activations for the beer and water conditions compared with the implicit baseline in the three phases of the BID task (continued)*

| Precentral Gyrus | L |  | -36 | 3 | 15 | 8.73 |  |
| --- | --- | --- | --- | --- | --- | --- | --- |
|  |  |  |  |  |  |  |  |
| **Postcentral Gyrus** | R | 3924 | 47 | -10 | 15 | 13.14 | 0.000 |
| Insula | R |  | 40 | -7 | 10 | 12.54 |  |
| Insula | R |  | 37 | -4 | 8 | 12.52 |  |
| Postcentral Gyrus | R |  | 52 | -7 | 12 | 12.27 |  |
| Postcentral Gyrus | R |  | 64 | -12 | 32 | 11.66 |  |
| Parietal Lobe | R |  | 67 | -17 | 28 | 11.64 |  |
| Postcentral Gyrus | R |  | 64 | -14 | 25 | 11.39 |  |
| Parietal Lobe | R |  | 64 | -24 | 32 | 10.92 |  |
| Parietal Lobe | R |  | 62 | -20 | 40 | 10.17 |  |
| Postcentral Gyrus | R |  | 64 | -2 | 8 | 9.97 |  |
| Superior Temporal Gyrus | R |  | 62 | 0 | 5 | 9.96 |  |
| Insula | R |  | 34 | 6 | 12 | 9.36 |  |
| Parietal Lobe | R |  | 60 | -22 | 45 | 9.05 |  |
| Postcentral Gyrus | R |  | 47 | -24 | 20 | 7.91 |  |
| Precentral Gyrus | R |  | 24 | -10 | 62 | 5.74 |  |
| Precentral Gyrus | R |  | 30 | -10 | 68 | 5.72 |  |
|  |  |  |  |  |  |  |  |
| **Nucleus Accumbens** | R | 1000 | 10 | 8 | -12 | 9.27 | 0.000 |
| Nucleus Accumbens | R |  | 12 | 10 | -10 | 8.94 |  |
| Anterior Cingulate Gyrus | R |  | 2 | 36 | 5 | 7.67 |  |
| Nucleus Accumbens | L |  | -10 | 6 | -12 | 7.61 |  |
| Anterior Cingulate Gyrus | R |  | 4 | 36 | 15 | 6.76 |  |
| Subgenual Anterior Cingulate Gyrus | L |  | -3 | 20 | -8 | 5.41 |  |
| Nucleus Accumbens | R |  | 7 | -4 | -12 | 4.77 |  |
| Nucleus Accumbens | L |  | -3 | -4 | -12 | 3.52 |  |
|  |  |  |  |  |  |  |  |
| **Thalamus** | L | 102 | -23 | -27 | -5 | 7.57 | 0.036 |
|  |  |  |  |  |  |  |  |
| **Posterior Cingulate Gyrus** | R | 501 | 12 | -34 | 40 | 6.65 | 0.000 |
| Superior Parietal Gyrus | R |  | 12 | -40 | 42 | 6.39 |  |

*Table S3: Whole brain activations for the beer and water conditions compared with the implicit baseline in the three phases of the BID task (continued)*

| Postcentral Gyrus | R |  | 12 | -34 | 45 | 6.32 |  |
| --- | --- | --- | --- | --- | --- | --- | --- |
| Postcentral Gyrus | R |  | 14 | -32 | 48 | 6.12 |  |
| Posterior Cingulate Gyrus | R |  | 7 | -12 | 48 | 5.76 |  |
| Superior Frontal Gyrus | R |  | 10 | -12 | 52 | 5.49 |  |
| Posterior Cingulate Gyrus | R |  | 7 | -14 | 55 | 5.49 |  |
|  |  |  |  |  |  |  |  |
| **Superior Parietal Gyrus** | R | 138 | 20 | -47 | 62 | 6.18 | 0.011 |
| Superior Parietal Gyrus | R |  | 30 | -37 | 52 | 3.94 |  |
|  |  |  |  |  |  |  |  |
| **Posterior Cingulate Gyrus** | L | 130 | -13 | -34 | 42 | 5.83 | 0.014 |
| Water |  |  |  |  |  |  |  |
| **Postcentral Gyrus** | L | 32356 | -53 | -12 | 32 | 33.97 | 0.000 |
| Postcentral Gyrus | R |  | 52 | -10 | 32 | 33.90 |  |
| Cerebellum | L |  | -16 | -62 | -20 | 33.50 |  |
| Precentral Gyrus | R |  | 57 | -4 | 28 | 32.08 |  |
| Precentral Gyrus | R |  | 60 | -2 | 25 | 32.07 |  |
| Postcentral Gyrus | R |  | 57 | -10 | 15 | 31.98 |  |
| Postcentral Gyrus | R |  | 37 | -7 | 15 | 30.77 |  |
| Cerebellum | R |  | 20 | -62 | -20 | 30.19 |  |
| Precentral Gyrus | L |  | -43 | -12 | 38 | 29.52 |  |
| Precentral Gyrus | L |  | -60 | -2 | 22 | 28.56 |  |
| Postcentral Gyrus | L |  | -58 | -10 | 12 | 27.74 |  |
| Precentral Gyrus | R |  | 62 | 8 | 22 | 27.73 |  |
| Postcentral Gyrus | L |  | -56 | -7 | 15 | 27.62 |  |
| Postcentral Gyrus | L |  | -26 | -2 | -10 | 26.67 |  |
| Postcentral Gyrus | L |  | -36 | -10 | 15 | 25.84 |  |
| Thalamus | R |  | 14 | -17 | 2 | 24.71 |  |
| Anterior Orbital Gyrus | R | 100 | 20 | 33 | -18 | 10.25 | 0.046 |
| Anterior Orbital Gyrus | L | 102 | -23 | 36 | -18 | 9.03 | 0.043 |
| Middle Frontal Gyrus | R | 195 | 44 | 46 | 5 | 8.07 | 0.002 |
|  |  |  |  |  |  |  |  |

*Table S3: Whole brain activations for the beer and water conditions compared with the implicit baseline in the three phases of the BID task (continued)*

| Beer |  |  |  |  |  |  |  |
| --- | --- | --- | --- | --- | --- | --- | --- |
| **Insula** | R | 9945 | 37 | -7 | 10 | 20.61 | 0.000 |
| Postcentral Gyrus | R |  | 57 | -7 | 12 | 18.53 |  |
| Postcentral Gyrus | R |  | 50 | -10 | 15 | 18.27 |  |
| Postcentral Gyrus | R |  | 44 | -7 | 18 | 18.15 |  |
| Postcentral Gyrus | L |  | -58 | -10 | 12 | 17.40 |  |
| Postcentral Gyrus | L |  | -46 | -10 | 20 | 17.32 |  |
| Insula | L |  | -36 | -10 | 12 | 17.25 |  |
| Postcentral Gyrus | L |  | -63 | -14 | 30 | 16.97 |  |
| Postcentral Gyrus | R |  | 64 | -12 | 32 | 16.77 |  |
| Postcentral Gyrus | L |  | -38 | -12 | 18 | 16.42 |  |
| Postcentral Gyrus | L |  | -63 | -14 | 20 | 16.22 |  |
| Insula | L |  | -38 | -4 | 2 | 14.89 |  |
| Precentral Gyrus | R |  | 62 | 0 | 25 | 14.28 |  |
| Insula | L |  | -38 | 3 | -8 | 13.85 |  |
| Insula | R |  | 34 | 8 | 12 | 11.43 |  |
| Nucleus Accumbens | R |  | 10 | 8 | -10 | 10.70 |  |
|  |  |  |  |  |  |  |  |
| **Cerebellum** | L | 4075 | -16 | -60 | -22 | 20.19 | 0.000 |
| Cerebellum | R |  | 17 | -62 | -20 | 18.82 |  |
| Lingual Gyrus | R |  | 17 | -87 | -8 | 17.50 |  |
| Occipital Lobe | L |  | -13 | -92 | -10 | 15.31 |  |
| Lingual Gyrus | L |  | -6 | -67 | 8 | 8.69 |  |
| Cuneus | L |  | -6 | -77 | 10 | 8.10 |  |
| Lingual Gyrus | R |  | 10 | -67 | 12 | 7.54 |  |
| Cuneus | L |  | -8 | -82 | 28 | 3.88 |  |
|  |  |  |  |  |  |  |  |
| **Thalamus** | R | 116 | 22 | -24 | -5 | 7.03 | 0.025 |
| Thalamus | R |  | 14 | -17 | 2 | 6.29 |  |
|  |  |  |  |  |  |  |  |
| **Middle Frontal Gyrus** | R | 108 | 44 | 43 | 8 | 6.30 | 0.033 |
|  |  |  |  |  |  |  |  |

*Table S3: Whole brain activations for the beer and water conditions compared with the implicit baseline in the three phases of the BID task (continued)*

| **Superior Parietal Gyrus** | R | 135 | 20 | -47 | 62 | 5.99 | 0.014 |
| --- | --- | --- | --- | --- | --- | --- | --- |
| Superior Parietal Gyrus | R |  | 32 | -37 | 52 | 4.18 |  |
|  |  |  |  |  |  |  |  |
| **Cerebellum** | L | 199 | -28 | -34 | -30 | 5.67 | 0.002 |
| Posterior Temporal Lobe | L |  | -38 | -44 | -20 | 4.53 |  |
| Fusiform Gyrus | L |  | -30 | -30 | -25 | 3.81 |  |
| Fusiform Gyrus | L |  | -38 | -32 | -22 | 3.64 |  |
| Water |  |  |  |  |  |  |  |
| **Lingual Gyrus** | R | 4657 | 17 | -87 | -8 | 18.64 | 0.000 |
| Occipital Lobe | L |  | -13 | -92 | -8 | 17.35 |  |
| Occipital Lobe | L |  | -28 | -80 | -12 | 14.67 |  |
| Cerebellum | R |  | 17 | -60 | -22 | 11.97 |  |
| Cerebellum | L |  | -16 | -60 | -22 | 11.91 |  |
| Lingual Gyrus | L |  | -6 | -67 | 8 | 8.21 |  |
| Posterior Temporal Lobe | L |  | -38 | -44 | -20 | 7.84 |  |
| Cuneus | L |  | -6 | -77 | 10 | 7.59 |  |
| Lingual Gyrus | R |  | 10 | -67 | 10 | 7.22 |  |
| Posterior Temporal Lobe | L |  | -38 | -54 | -15 | 6.36 |  |
| Superior Parietal Gyrus | R |  | 20 | -57 | 20 | 5.86 |  |
| Superior Parietal Gyrus | L |  | -13 | -60 | 20 | 5.66 |  |
| Fusiform Gyrus | L |  | -38 | -22 | -25 | 3.72 |  |
| Cuneus | L |  | -8 | -80 | 28 | 3.67 |  |
|  |  |  |  |  |  |  |  |
| **Postcentral Gyrus** | L | 3185 | -38 | -12 | 22 | 13.57 | 0.000 |
| Postcentral Gyrus | L |  | -66 | -17 | 25 | 12.48 |  |
| Postcentral Gyrus | L |  | -58 | -12 | 15 | 11.33 |  |
| Postcentral Gyrus | L |  | -38 | -12 | 22 | 13.57 |  |
| Postcentral Gyrus | L |  | -66 | -17 | 25 | 12.48 |  |
| Postcentral Gyrus | L |  | -58 | -12 | 15 | 11.33 |  |
| Insula | L |  | -36 | -10 | 12 | 11.18 |  |
| Insula | L |  | -38 | 0 | -8 | 10.57 |  |

*Table S3: Whole brain activations for the beer and water conditions compared with the implicit baseline in the three phases of the BID task (continued)*

| Parietal Lobe | L |  | -66 | -27 | 25 | 10.38 |  |
| --- | --- | --- | --- | --- | --- | --- | --- |
| Parietal Lobe | L |  | -63 | -30 | 28 | 10.37 |  |
| Precentral Gyrus | L |  | -58 | -7 | 38 | 10.00 |  |
| Precentral Gyrus | L |  | -36 | 3 | 15 | 8.73 |  |
|  |  |  |  |  |  |  |  |
| **Postcentral Gyrus** | R | 3924 | 47 | -10 | 15 | 13.14 | 0.000 |
| Insula | R |  | 40 | -7 | 10 | 12.54 |  |
| Insula | R |  | 37 | -4 | 8 | 12.52 |  |
| Postcentral Gyrus | R |  | 52 | -7 | 12 | 12.27 |  |
| Postcentral Gyrus | R |  | 64 | -12 | 32 | 11.66 |  |
| Parietal Lobe | R |  | 67 | -17 | 28 | 11.64 |  |
| Postcentral Gyrus | R |  | 64 | -14 | 25 | 11.39 |  |
| Parietal Lobe | R |  | 64 | -24 | 32 | 10.92 |  |
| Parietal Lobe | R |  | 62 | -20 | 40 | 10.17 |  |
| Postcentral Gyrus | R |  | 64 | -2 | 8 | 9.97 |  |
| Superior Temporal Gyrus | R |  | 62 | 0 | 5 | 9.96 |  |
| Insula | R |  | 34 | 6 | 12 | 9.36 |  |
| Postcentral Gyrus | R |  | 60 | -22 | 45 | 9.05 |  |
| Parietal Lobe | R |  | 47 | -24 | 20 | 7.91 |  |
| Precentral Gyrus | R |  | 24 | -10 | 62 | 5.74 |  |
| Precentral Gyrus | R |  | 30 | -10 | 68 | 5.72 |  |
|  |  |  |  |  |  |  |  |
| **Nucleus Accumbens** | R | 1000 | 10 | 8 | -12 | 9.27 | 0.000 |
| Nucleus Accumbens | R |  | 12 | 10 | -10 | 8.94 |  |
| Anterior Cingulate Cortex | R |  | 2 | 36 | 5 | 7.67 |  |
| Nucleus Accumbens | L |  | -10 | 6 | -12 | 7.61 |  |
| Anterior Cingulate cortex | R |  | 4 | 36 | 15 | 6.76 |  |
| Subgenual anterior cingulate cortex | L |  | -3 | 20 | -8 | 5.41 |  |
| Nucleus Accumbens | R |  | 7 | -4 | -12 | 4.77 |  |
| Nucleus Accumbens | L |  | -3 | -4 | -12 | 3.52 |  |
|  |  |  |  |  |  |  |  |
| **Thalamus** | L | 102 | -23 | -27 | -5 | 7.57 | 0.036 |

*Table S3: Whole brain activations for the beer and water conditions compared with the implicit baseline in the three phases of the BID task (continued)*

|  |  |  |  |  |  |  |  |  |
| --- | --- | --- | --- | --- | --- | --- | --- | --- |
| **Posterior Cingulate Gyrus** | R | 501 | 12 | -34 | 40 | 6.65 | 0.000 |  |
| Superior Parietal Gyrus | R |  | 12 | -40 | 42 | 6.39 |  |  |
| Postcentral Gyrus | R |  | 12 | -34 | 45 | 6.32 |  |  |
| Postcentral Gyrus | R |  | 14 | -32 | 48 | 6.12 |  |  |
| Posterior Cingulate Gyrus | R |  | 7 | -12 | 48 | 5.76 |  |  |
| Superior Frontal Gyrus | R |  | 10 | -12 | 52 | 5.49 |  |  |
| Superior Frontal Gyrus | R |  | 7 | -14 | 55 | 5.49 |  |  |
| Superior Parietal Gyrus | R | 138 | 20 | -47 | 62 | 6.18 | 0.011 |  |
| Posterior Cingulate Gyrus | R |  | 30 | -37 | 52 | 3.94 |  |  |
|  |  |  |  |  |  |  |  |  |
| **Posterior Cingulate Gyrus** | L | 130 | -13 | -34 | 42 | 5.83 | 0.014 |  |
|  |  |  |  |  |  |  |  |  |
| *Note:* Reported coordinates correspond to all peak voxels more than 4 mm apart in each cluster P-values are cluster level FWE-corrected | | | | | | | | |
